# Supplementary material for: Unravelling the skills of data scientists: A text mining analysis of Dutch university master programs in data science and artificial intelligence
Source: PLoS One. 2024 Feb 29;19(2):e0299327. doi: 10.1371/journal.pone.0299327 (PMC10903789; doi:10.1371/journal.pone.0299327)
Supplement: S1 Appendix — (DOCX) [file pone.0299327.s001.docx]

**Appendix A**

**Search keys Data Science and Artificial Intelligence**

- knowledge map AND data science
- knowledge map AND data science OR AI OR artificial intelligence
- knowledge map AND artificial intelligence
- knowledge map AND computer science
- knowledge map AND data science OR AI OR artificial intelligence OR computer science
- definition of data science
