# Supplementary material for: Unravelling the skills of data scientists: A text mining analysis of Dutch university master programs in data science and artificial intelligence
Source: PLoS One. 2024 Feb 29;19(2):e0299327. doi: 10.1371/journal.pone.0299327 (PMC10903789; doi:10.1371/journal.pone.0299327)
Supplement: S2 Appendix — (DOCX) [file pone.0299327.s002.docx]

**Appendix B**

**Erasing non-informative words: “ad” and “hoc”**

The words “ad” and “hoc got printed in one of topics of the LSA model. The words “Ad”, “hoc” and “ad_hoc” would be stated here, which would contain 30% of the total shown words per topic, whereas the words would not hold any interpretable information. The words ad and hoc were only used in one description, but were used a lot and always together. This would lead to the LSA prioritizing this word to the extent where it would end up in one of the top 10 words of one of the 7 topics. This would lead to too much information being lost and therefore we decided to delete the words from the descriptions. Therefore the optimal number of K metrics and all analyses were rerun. Since the algorithms for finding an optimal number of K would run for two full days on a normal computer with a quad core processor. In light of not spending too much time we chose to ignore other combinations of words that would lead to some information being lost. An example of this case is in the LSA where k = 7 outcomes, topic 4 where “multimedia”, “search” and “multimedia_search” would be stated. These cases erose after removing the words “ad” and “hoc” and therefore this might become an endless cycle of removing non-optimal words from the analyses.
