# Supplementary material for: Unravelling the skills of data scientists: A text mining analysis of Dutch university master programs in data science and artificial intelligence
Source: PLoS One. 2024 Feb 29;19(2):e0299327. doi: 10.1371/journal.pone.0299327 (PMC10903789; doi:10.1371/journal.pone.0299327)
Supplement: S3 Appendix — (DOCX) [file pone.0299327.s003.docx]

**Appendix C**

**Top ten most frequent words per topic for the CTM with 13 Topics**

|  | Topic 1 | Topic 2 | Topic 3 | Topic 4 | Topic 5 | Topic 6 | Topic 7 |
| --- | --- | --- | --- | --- | --- | --- | --- |
| 1 | network | model | data | data | data | system | research |
| 2 | datascience | data | process | techniques | information | problem | design |
| 3 | programme | statistical | naturallanguageprocessing | algorithms | model | algorithms | scientific |
| 4 | data | analysis | techniques | analysis | programming | quantum | project |
| 5 | social | techniques | model | machinelearning | visualization | techniques | knowledge |
| 6 | research | methods | web | datamining | system | optimization | field |
| 7 | knowledge | theory | language | information | retrieval | artificialintelligence | researchproject |
| 8 | artificialintelligence | linear | datascience | knowledge | language | design | software |
| 9 | project | timeseries | information | theory | processing | methods | speech |
| 10 | information | regression | mining | applications | big | model | questions |
| Core | *Core terms* | *Statistical analyses* | *Natural language processing* | *Machine learning* | *Information* | *AI / optimization* | *Research* |

|  | Topic 8 | Topic 9 | Topic 10 | Topic 11 | Topic 12 | Topic 13 |
| --- | --- | --- | --- | --- | --- | --- |
| 1 | image | system | deeplearning | business | project | model |
| 2 | machinelearning | design | reinforcement | data | research | system |
| 3 | model | security | algorithms | marketing | thesis | computational |
| 4 | data | data | model | datascience | economic | methods |
| 5 | techniques | health | neuronetwork | innovation | system | modeling |
| 6 | methods | business | system | management | analysis | human |
| 7 | analysis | management | methods | services | problem | processes |
| 8 | processing | software | deep | knowledge | data | theoretical |
| 9 | computer | digital | problem | concepts | services | neuroscience |
| 10 | vision | information | techniques | customer | start | cognitive |
| Core | Analyses | Information security / business | Machine learning | Business domain | Research | Modeling / Human brain |
