# Supplementary material for: Unravelling the skills of data scientists: A text mining analysis of Dutch university master programs in data science and artificial intelligence
Source: PLoS One. 2024 Feb 29;19(2):e0299327. doi: 10.1371/journal.pone.0299327 (PMC10903789; doi:10.1371/journal.pone.0299327)
Supplement: S4 Appendix — (DOCX) [file pone.0299327.s004.docx]

**Appendix D**

**Overview of 2021 master programs: distribution by institution and corpus of student body by enrollment numbers, categorized into general (G) or specific (S) programs**

|  | ***Institution*** | ***Program*** | ***Corpus*** | ***Type*** |
| --- | --- | --- | --- | --- |
| 1 | University of Groningen (RUG) | Artificial intelligence | 204 | G |
| 2 | University of Groningen (RUG) | MSc Marketing - Marketing Analytics and Data Science (master track Marketing) | 350* | S |
| 3 | Vrije Universiteit Amsterdam (VU) | Artificial intelligence | 357 | G |
| 4 | Vrije Universiteit (VU) | Master programme in Econometrics and Operations Research: Econometrics and Data Science specialization track (MSc) | 367* | S |
| 5 | Vrije Universiteit (VU) | Master programme in Econometrics and Operations Research: Marketing Data Science specialization track (MSc) | 367* | S |
| 6 | Vrije Universiteit (VU) | Big data Engineering (track Computer science) | 208* | G |
| 7 | Vrije Universiteit (VU) (and Erasmus University of Rotterdam and UVA) | Research Master Business Data Science | n..a | S |
| 8 | University of Amsterdam (UVA) | Artificial intelligence | 359 | G |
| 9 | University of Amsterdam (UVA) | Data Analytics (MSc Econometrics) | 155 | SS |
| 10 | University of Amsterdam (UVA) | Information Studies: Data Science (track) | 251* | G |
| 11 | University of Amsterdam (UVA) | MBA Big Data & Business Analytics | n.a | S |
| 12 | University of Amsterdam (UVA) | Psychology: Behavioral Data Science (track) | 357 | S |
| 13 | Leiden University (LU) | Statistics and Data Science | 154 | G |
| 14 | Leiden University (LU) | Data Science track: Computer Science (MSc) | 286* | G |
| 15 | Leiden University (LU) | Astronomy and Data Science, MSc (track Astronomy) | 119* | S |
| 16 | Delft University of Technology (TU Delft) | Computer Science -> Data Science & Technology (track Computer science) | 724* | G |
| 17 | Eindhoven University of Technology (TU/e) | Master Data Science and Entrepreneurship (JADS) | 79 | S |
| 18 | Eindhoven University of Technology (TU/e) | Master Data Science and Artificial Intelligence | 135 | G |
| 19 | Eindhoven University of Technology (TU/e) | Smart Mobility Data Science and Analytics (SMDA) (track Architecture, Building and Planning) | 537* | S |
| 20 | Eindhoven University of Technology (TU/e) | EngD Data Science (previously PdEng) | n.a | G |
| 21 | Eindhoven University of Technology (TU/e) | Special Master track EIT Digital Data Science (International double degree program) | n.a | G |
| 22 | Tilburg University (TiL) | Data Science and Society (JADS) (MSc) | 428* | S |
| 23 | Tilburg University (TiL) | Economics: Data Science (track Economics) | 145 | S |
| 24 | Tilburg University (TiL) | Business Analytics and Operations Research (MSc) | 106 | S |
| 25 | Tilburg University (TiL) | Marketing Analytics (MSc) | 140 | S |
| 26 | Tilburg University (TiL) | Cognitive Science and Artificial Intelligence (MSc) | <17 | S |
| 27 | Utrecht University (UU) | Applied data science | 177 | G |
| 28 | Utrecht University (UU) | Artificial intelligence | 293 | G |
| 29 | University of Twente (UT) | Computer Vision and Biometrics (track Electrical Engineering) | 220 | S |
| 30 | University of Twente (UT) | Data Science (EIT Digital double degree) | n.a | G |
| 31 | University of Twente (UT) | Data Science & Business (track Business Information Technology) | 114* | S |
| 32 | University of Twente (UT) | Data Science & Technology (track Business Information Technology) | 114* | G |
| 33 | University of Twente (UT) | Mathematics of Data Science (track Applied Mathematics) | 83* | G |
| 34 | University of Twente (UT) | Sports Data Science (track Computer Science) | 300* | S |
| 35 | Maastricht University (MU) | Data Science for Decision Making | n.a | S |
| 36 | Maastricht University (MU) | Artificial intelligence | n.a | G |
| 37 | Maastricht University (MU) | Business Intelligence and Smart Service | 59 | S |
| 38 | Maastricht University (MU) | Digital Business and Economics | <14 | S |
| 39 | Maastricht University (MU) | Systems Biology | 34 | S |
| 40 | Radboud University Nijmegen (RAD) | Artificial intelligence | 180 | G |
| 41 | Radboud University Nijmegen (RAD) | Master's specialization: Data Science (track Computing Science) | 351* | G |

For specialization tracks within a broader program, the corpus is available exclusively for the full program. The corpus is extracted from the official statistics provided by DUO, the agency responsible for administering educational services in the Netherlands: <https://duo.nl/open_onderwijsdata/hoger-onderwijs/aantal-studenten/studenten-wo.jsp>
