# Supplementary material for: Unravelling the skills of data scientists: A text mining analysis of Dutch university master programs in data science and artificial intelligence
Source: PLoS One. 2024 Feb 29;19(2):e0299327. doi: 10.1371/journal.pone.0299327 (PMC10903789; doi:10.1371/journal.pone.0299327)
Supplement: S5 Appendix — (DOCX) [file pone.0299327.s005.docx]

**Appendix E**

**The posterior distribution of the 7 topics across the 4 master programs offered at Utrecht (UU, top) and Groningen University (RUG, bottom).**
