# Supplementary material for: Unravelling the skills of data scientists: A text mining analysis of Dutch university master programs in data science and artificial intelligence
Source: PLoS One. 2024 Feb 29;19(2):e0299327. doi: 10.1371/journal.pone.0299327 (PMC10903789; doi:10.1371/journal.pone.0299327)
Supplement: S6 Appendix — (DOCX) [file pone.0299327.s006.docx]

|  | Course/program name | Program | University | Core terms | Statistics | DPT_3 | DPT_4 | Electronics/IT | Ethics | Research |
| --- | --- | --- | --- | --- | --- | --- | --- | --- | --- | --- |
| 1 | AI | AI | RUG | 0.30949885671319 | 9.67288581513981e-05 | 8.48855762098098e-06 | 0.15620257378071 | 0.000198335603708243 | 0.405579728760796 | 0.128415287725824 |
| 2 | Design of Multi-Agent Systems | AI | RUG | 0.00589767169263515 | 0.00131082424449539 | 0.00011623479156368 | 0.002098353729517 | 0.000898254048966023 | 0.488505388490409 | 0.501173273002414 |
| 3 | Machine Learning | AI | RUG | 0.000122940064833071 | 0.122513094245388 | 0.151866441871773 | 0.566030231781456 | 0.000106935422758837 | 9.55975801746715e-05 | 0.159264759033617 |
| 4 | Deep Learning | AI | RUG | 0.000605884003747479 | 0.000273455733828095 | 0.964004864752965 | 0.00033526987368317 | 0.000153242077289345 | 8.46731078016714e-06 | 0.0346188162477064 |
| 5 | Final Research Project AI | AI | RUG | 0.934959604624127 | 7.67321441944218e-05 | 0.000116758358174004 | 4.52026714503501e-06 | 0.000173913417073909 | 0.000591716260005043 | 0.0640767549292804 |
| 6 | Cognitive Robotics | AI | RUG | 0.00139069610336009 | 0.000149385072090969 | 4.35945011461214e-06 | 0.000364747116423925 | 0.000175044828735109 | 0.91602499481857 | 0.0818907726107056 |
| 7 | Pattern Recognition | AI | RUG | 0.000263633825195627 | 0.915338634198501 | 0.0427755685660584 | 0.000618050083091221 | 1.70894482733025e-05 | 1.73410316020583e-05 | 0.0409696828472778 |
| 8 | Handwriting Recognition | AI | RUG | 0.000236139865741098 | 0.246600995307769 | 0.360862577691119 | 0.208128424942704 | 0.000131215496701334 | 8.67999081686178e-05 | 0.183953846787797 |
| 9 | Arguing Agents | AI | RUG | 0.00522475194942066 | 0.000597654593930184 | 4.33400927165469e-05 | 0.00126015628453393 | 0.000584350172934203 | 0.686522953666319 | 0.305766793240146 |
| 10 | Computational Social Choice | AI | RUG | 0.0030023457163324 | 0.103363842092225 | 0.00189629517652763 | 0.0663700198138357 | 0.000583148333392642 | 0.00385841378976008 | 0.820925935077926 |
| 11 | Logical Aspects of Multi-agent Systems | AI | RUG | 0.000126109807722281 | 0.000117204797356205 | 2.06006235967981e-05 | 0.690371782203973 | 0.0883129532264065 | 0.143223809939071 | 0.0778275394018745 |
| 12 | Cognitive Robotics | AI | RUG | 0.00139069610336009 | 0.000149385072090969 | 4.35945011461214e-06 | 0.000364747116423925 | 0.000175044828735109 | 0.91602499481857 | 0.0818907726107056 |
| 13 | Pattern Recognition | AI | RUG | 0.000421401455805335 | 0.889244280043112 | 0.045934815496681 | 0.000769298646472975 | 2.72932601310782e-05 | 3.2334465651356e-05 | 0.0635705766321461 |
| 14 | Robotics for AI | AI | RUG | 0.000263904243002445 | 0.113837571284623 | 1.18302016113274e-05 | 0.119613559645909 | 7.27440884653743e-05 | 0.614726685321727 | 0.151473705214661 |
| 15 | Introduction Science and Policy | AI | RUG | 0.973119104434245 | 2.74899549815548e-05 | 4.77528664938445e-05 | 9.75520367198596e-07 | 0.000105430454102317 | 0.000344486630566508 | 0.0263547601392438 |
| 16 | Introduction Science and Business | AI | RUG | 0.978530449062363 | 2.09829521807417e-05 | 3.78379345294324e-05 | 6.53158402368084e-07 | 9.55287191852829e-05 | 0.000303715326796786 | 0.021010832846542 |
| 17 | Skills in Science Communication | AI | RUG | 0.817993443347557 | 0.000230485780271028 | 0.000312649025425524 | 2.57393312116152e-05 | 0.000336977240152762 | 0.0014781121111486 | 0.179622593164234 |
| 18 | Auditory and Visual Perception | AI | RUG | 0.00554838238865384 | 0.161286595521546 | 0.265092812552662 | 0.00238049331457064 | 0.000399161607352861 | 0.000413975467346462 | 0.564878579147868 |
| 19 | Cognitive Engineering | AI | RUG | 0.00893341292295773 | 0.00124512257515486 | 0.000490627068779705 | 0.00160980644954793 | 0.00118480793163528 | 0.0820855398118209 | 0.904450683240104 |
| 20 | Introduction to Data Science | AI | RUG | 0.103453724946851 | 0.00131220775980838 | 0.243327726766993 | 0.110389243094894 | 0.000577044766741444 | 0.000428308327203394 | 0.540511744337508 |
| 21 | Language Modelling | AI | RUG | 0.00549933796597582 | 0.00229953921560349 | 0.0464646989936448 | 0.00191517379643446 | 0.000834463790098127 | 0.00117990019704466 | 0.941806886041199 |
| 22 | Robotics for IEM | AI | RUG | 0.00308720208675898 | 0.102145629300155 | 0.000202464947575267 | 0.00188286049782259 | 0.0373370057733493 | 0.113901515228189 | 0.74144332216615 |
| 23 | Semantic Web Technology | AI | RUG | 0.0513109602401609 | 0.000163640200274607 | 0.885974872947012 | 4.21887196092519e-05 | 0.000244510711400338 | 1.78143579511905e-05 | 0.0622460128235911 |
| 24 | User Models | AI | RUG | 0.00918947760500489 | 0.00368694993412101 | 0.00205239792862186 | 0.0028939269379617 | 0.00150005870938986 | 0.00534115024314991 | 0.975336038641751 |
| 25 | Web and Cloud Computing | AI | RUG | 0.000256454242433198 | 0.000149071100953897 | 0.000428960921407057 | 0.161487719036654 | 0.738648644869426 | 0.000782134939735306 | 0.0982470148893909 |
| 26 | Advanced Computer Graphics | AI | RUG | 0.00167620942676006 | 0.00133512863666757 | 0.00180295279576618 | 0.327164825309754 | 0.0441993327884517 | 0.00248708376326779 | 0.621334467279332 |
| 27 | Cognitive Modelling: Basic Principles and Methods | AI | RUG | 0.256175920597063 | 0.148671777957368 | 0.00102735726501757 | 0.00031486956390149 | 0.000307678816860851 | 0.00181682488438123 | 0.591685570915408 |
| 28 | Computational Semantics | AI | RUG | 0.0005696231777238 | 0.0013641506907218 | 0.629455530463739 | 0.147018117622804 | 0.000462749784050115 | 0.000153561241984646 | 0.220976267018977 |
| 29 | Neural Networks and Computational Intelligence | AI | RUG | 0.000655374114764481 | 0.00119647892739918 | 0.254067831867466 | 0.359346813696722 | 0.000508472529790418 | 0.0208450014734745 | 0.363380027390383 |
| 30 | Scientific Visualization | AI | RUG | 8.76858015949422e-05 | 9.49343165438925e-05 | 0.000248022082057195 | 0.404640196932304 | 0.5360364795812 | 0.000603767778495664 | 0.0582889135078041 |
| 31 | Applied Cognitive Engineering | AI | RUG | 0.00981424473794819 | 0.000902261277571979 | 0.000132062394533194 | 0.0017006314644486 | 0.0011244167987902 | 0.421312751410231 | 0.565013631916477 |
| 32 | Auditory Biophysics | AI | RUG | 0.000203085440358578 | 0.321714500731471 | 0.645229848232828 | 0.00048126496534549 | 1.83116962198021e-05 | 3.41084038233996e-06 | 0.0323495780933948 |
| 33 | Cognitive Modelling: Complex Behaviour | AI | RUG | 0.384061171726768 | 0.000435015036543577 | 0.000974441961460436 | 0.000203337410941949 | 0.0219991633466805 | 0.00253618222929032 | 0.589790688288316 |
| 34 | Computational Simulations of Language | AI | RUG | 0.024532499851323 | 0.278032143655201 | 0.281789201449329 | 0.000654877006162595 | 0.000181228606851472 | 0.000183444693646216 | 0.414626604737486 |
| 35 | Computer Vision | AI | RUG | 5.88564668467118e-05 | 0.858381309726262 | 0.000216652535833048 | 0.0918862948635032 | 1.99769882837959e-05 | 0.000247578993845655 | 0.0491893304254252 |
| 36 | Fundamentals of Distributed System | AI | RUG | 0.000111498653493619 | 7.11846637664745e-05 | 0.000207931505929985 | 0.142419734969133 | 0.803495681814544 | 0.00050831571685602 | 0.0531856526762764 |
| 37 | Natural Language Processing | AI | RUG | 2.9174720729931e-05 | 0.000642778526655055 | 0.437472796641305 | 0.527053324829571 | 0.000146113003073901 | 1.61448764988452e-05 | 0.0346396674021664 |
| 38 | Advanced self-organisation of social systems | AI | RUG | 0.145290963134137 | 0.0566047165067346 | 0.0012000395673749 | 0.00065569866258994 | 0.000438598596460612 | 0.00321361962663246 | 0.792596363906071 |
| 39 | Computational Cognitive Neuroscience | AI | RUG | 0.00939632082776595 | 0.166788159550751 | 0.00168252750294375 | 0.002356912057711 | 0.00052606788804784 | 0.00270333327319073 | 0.816546678899589 |
| 40 | Language Technology Project | AI | RUG | 0.158491175373307 | 0.000578384841853438 | 0.52778471059332 | 0.00020011031804629 | 0.000650380608646888 | 0.00021716063556918 | 0.312078077629257 |
| 41 | Neuro-ergonomics | AI | RUG | 0.00658536740800994 | 0.0012405539484043 | 0.00231082719076955 | 0.00345877036930786 | 0.069823974127372 | 0.00476081791597241 | 0.911819689040164 |
| 42 | MA&DS | MA&DS | RUG | 0.701007654306176 | 0.000405585656438654 | 0.00050867994200602 | 6.11091539461737e-05 | 0.000568456660915274 | 0.00175584084786746 | 0.29569267343265 |
| 43 | Companies, Brands, and Consumers | MA&DS | RUG | 0.00296328443218596 | 0.000261481532166764 | 1.35659530994773e-05 | 0.000517556626254491 | 0.000288703331391508 | 0.83359676475812 | 0.162358643366781 |
| 44 | Data Engineering for MADS | MA&DS | RUG | 0.00256190498053272 | 0.000829911172188704 | 0.033770616510677 | 0.111280595224255 | 0.175949575096054 | 0.00118949121213348 | 0.674417905804159 |
| 45 | Statistical Learning in Marketing | MA&DS | RUG | 0.000786516098860072 | 0.281993724024324 | 0.000951777121679069 | 0.260849118515571 | 0.000226258456592276 | 0.00202380382474356 | 0.45316880195823 |
| 46 | Data Science Methods for MADS | MA&DS | RUG | 0.00968201238290409 | 0.00165650859511324 | 0.184677149051814 | 0.00211267443065693 | 0.00146615033761849 | 0.00092827211024305 | 0.79947723309165 |
| 47 | Retail & Omnichannel Marketing | MA&DS | RUG | 0.0477753407375371 | 0.000206222794067591 | 1.44573794161164e-05 | 0.000190456553487862 | 0.000353005725970868 | 0.754205424522473 | 0.197255092287048 |
| 48 | Market Models | MA&DS | RUG | 0.0050820598541774 | 0.0814412112803236 | 0.0015993842151929 | 0.00217522841592072 | 0.000563509452294782 | 0.00249417557865548 | 0.906644431203435 |
| 49 | Master's Thesis Marketing MADS | MA&DS | RUG | 0.606149942551546 | 0.000612524709970749 | 0.000728659362166974 | 0.000100150470891267 | 0.000569731432562821 | 0.0019885007100871 | 0.389850490762775 |
| 50 | Customer Management | MA&DS | RUG | 0.00521940119282533 | 0.000537807811622125 | 7.16753948925605e-05 | 0.000936093935717397 | 0.000599798726737789 | 0.550150385931361 | 0.442484837006843 |
| 51 | Digital Marketing Intelligence | MA&DS | RUG | 0.00817370683319184 | 0.00172420496833841 | 0.0143239096948026 | 0.00308326954396951 | 0.00109545211962103 | 0.187176367619339 | 0.784423089220737 |
| 52 | B2B Marketing | MA&DS | RUG | 0.123477854027567 | 1.41477065849208e-05 | 6.08206467849143e-06 | 4.12654561149389e-05 | 0.251879136643996 | 0.510192890729973 | 0.114388623371086 |
| 53 | Customer Models | MA&DS | RUG | 0.00564052389833891 | 0.0854769903781192 | 0.00160953389268411 | 0.00215960097009034 | 0.0005874213791904 | 0.00267191763850734 | 0.90185401184307 |
| 54 | APPLIED DATA SCIENCE | ADS | UU | 0.755132831208223 | 0.000110866921418578 | 0.142776180543635 | 1.03595705274634e-05 | 0.000204019061288084 | 6.40027913687446e-05 | 0.101701739903539 |
| 55 | DATA WRANGLING AND DATA ANALYSIS | ADS | UU | 0.0432829146455121 | 0.000649344053027024 | 0.00171767327738211 | 0.000939013682846432 | 0.191153185264143 | 0.00325423864299799 | 0.759003630434092 |
| 56 | COLLOQUIUM APPLIED DATA SCIENCE | ADS | UU | 0.775119819826576 | 0.000121997689799989 | 2.98335950206485e-05 | 2.35100095568723e-05 | 0.000352583898011859 | 0.0749814295527515 | 0.149370825428283 |
| 57 | EPIDEMIOLOGY AND BIG DATA | ADS | UU | 0.00302365341301064 | 0.719799225250667 | 0.000748239908261681 | 0.00132009702035033 | 0.000140058132146753 | 0.00110866320182627 | 0.273860063073738 |
| 58 | SPATIAL DATA ANALYSIS AND SIMULATION MODELLING | ADS | UU | 0.00136330783522378 | 0.852909351817601 | 0.000542709445935416 | 0.00154732899740413 | 8.0724123422236e-05 | 0.000771829932841878 | 0.142784747847571 |
| 59 | SOCIAL BEHAVIOUR DYNAMICS | ADS | UU | 0.0318090613906497 | 0.755019234439804 | 0.000222880663722906 | 0.000165432609184032 | 4.92014444019421e-05 | 0.000979650128388523 | 0.211754539323849 |
| 60 | NETWORK ANALYSIS | ADS | UU | 0.00275628282084433 | 0.0219994383091747 | 0.185758422913797 | 0.00283003516969491 | 0.000522795499157569 | 0.000607951698856765 | 0.785525073588475 |
| 61 | DATA MINING: TEXT, IMAGES, VIDEO | ADS | UU | 9.2744226460764e-05 | 0.00013469565191673 | 0.000362605942188181 | 0.398773345793894 | 0.544739476398125 | 0.000696198991237877 | 0.0552009329961784 |
| 62 | ARTIFICAL INTELLIGENCE | AI | UU | 0.307346517647588 | 0.000150181156648487 | 2.24301528391216e-05 | 0.152008361613809 | 0.000442885052015491 | 0.328082757027536 | 0.211946867349564 |
| 63 | METHODS IN AI RESEARCH | AI | UU | 0.00852798900831178 | 0.000996390656678755 | 0.000204544611498175 | 0.0978723320850149 | 0.000780463979455068 | 0.252144868377679 | 0.639473411281362 |
| 64 | PHILOSOPHY OF A.I. | AI | UU | 0.416572383493571 | 0.00068179200115921 | 0.000679951289684066 | 0.000197495408420943 | 0.000710713044905639 | 0.00558642355584648 | 0.575571241206412 |
| 65 | INTRODUCING NATURAL SCIENCES | AI | UU | 0.60089552634237 | 0.000673500407054958 | 0.000765045388452059 | 0.000121633367124404 | 0.000855451194349516 | 0.0032142084204816 | 0.393474634880167 |
| 66 | DILEMMAS OF THE SCIENTIST | AI | UU | 0.971284263706944 | 3.11975636229717e-05 | 5.40288445361895e-05 | 1.12383350227861e-06 | 0.000126279921954262 | 0.000389088103930898 | 0.0281140180255096 |
| 67 | INTELLIGENT AGENTS | AI | UU | 0.00161111981299071 | 2.41646977337702e-05 | 2.71616866067039e-06 | 0.000353903917591944 | 0.13809040768227 | 0.800798557562906 | 0.0591191301578467 |
| 68 | MACHINE LEARNING FOR HUMAN VISION AND LANGUAGE | AI | UU | 0.00386066911625389 | 0.00144597112513053 | 0.439086360430027 | 0.00171216584614955 | 0.000932133863415902 | 0.000432731208807175 | 0.552529968410216 |
| 69 | COMPUTATIONAL ARGUMENTATION | AI | UU | 0.00266650813943114 | 0.000333333882347983 | 1.3097077391694e-05 | 0.000694723165617906 | 0.000320790976171 | 0.839528412341716 | 0.156443134417324 |
| 70 | DATA MINING | AI | UU | 0.000584671636821925 | 0.00246461406423647 | 0.00135182558448981 | 0.771039830439057 | 0.000715225334883539 | 0.00228820055863078 | 0.22155563238188 |
| 71 | LOGIC AND LANGUAGE | AI | UU | 0.00180878361887667 | 0.0387162066324721 | 0.334755048014999 | 0.207240133171929 | 0.000329986216693917 | 0.000284420931587199 | 0.416865421413442 |
| 72 | ADVANCED MACHINE LEARNINGADVANCED MACHINE LEARNING | AI | UU | 4.74142707372175e-05 | 0.350956943059386 | 1.07372306767203e-05 | 0.476630806174436 | 2.59515234904317e-05 | 0.107590318952795 | 0.0647378287884789 |
| 73 | COGNITIVE MODELING | AI | UU | 0.0021870645071724 | 0.0411073591752335 | 0.000963349759460416 | 0.0633655237847605 | 0.000519500985783157 | 0.0183359775928545 | 0.873521224194736 |
| 74 | PATTERN RECOGNITION | AI | UU | 9.93037893447841e-06 | 0.428834426805095 | 0.00018754614635607 | 0.546392864298358 | 1.54220442391223e-05 | 0.000175015809891729 | 0.0243847945171253 |
| 75 | LOGIC AND COMPUTATION | AI | UU | 0.134022223128925 | 0.00116246516188398 | 0.0013651313558919 | 0.152248468622519 | 0.000949580712871264 | 0.0037543390281953 | 0.706497791989714 |
| 76 | MULTI-AGENT SYSTEMS | AI | UU | 0.00543693824917829 | 0.000772615619972613 | 5.50021424891574e-05 | 0.00148834952306692 | 0.000773184563665311 | 0.662164936350585 | 0.329308973551043 |
| 77 | EXPERIMENTATION IN PSYCHOLOGY, LINGUISTICS, AND AI | AI | UU | 0.00543365818455848 | 0.00197608282342419 | 0.0286111272125476 | 0.00216362486645961 | 0.000879842798236565 | 0.0014397306576933 | 0.95949593345708 |
| 78 | EVOLUTIONARY COMPUTING | AI | UU | 0.00023608351910932 | 0.137106663687902 | 0.000786960299582818 | 0.682802221722099 | 0.000178404998805671 | 0.00140701095454485 | 0.177482654817956 |
| 79 | SOCIAL COMPUTING | AI | UU | 0.0723341098797002 | 0.000934937849919643 | 0.00174720093546421 | 0.00111335479929097 | 0.0810706351389357 | 0.00406569400765771 | 0.838734067389032 |
| 80 | MULTI-AGENT LEARNING | AI | UU | 0.000952089232837393 | 0.0013638083296709 | 9.91297764126833e-05 | 0.447410195968096 | 0.000818043578160203 | 0.276031708511017 | 0.273325024603806 |
| 81 | NATURAL LANGUAGE PROCESSING | AI | UU | 0.000550447309188071 | 0.000251933947785016 | 0.968535483480956 | 0.000309495039931056 | 0.000150181258073842 | 6.86861675507271e-06 | 0.0301955903473113 |
| 82 | LOGICS FOR SAFE ARTIFICIAL INTELLIGENCE | AI | UU | 0.0015513868891548 | 0.16708625339574 | 8.12745300997423e-06 | 0.00107212296924082 | 6.9976735695176e-05 | 0.686305574255959 | 0.1439065583012 |
| 83 | HUMAN CENTERED MACHINE LEARNING | AI | UU | 0.0356817472744451 | 0.000547443302234885 | 0.0142482585823715 | 0.130561282632099 | 0.000813506298983968 | 0.292905389879713 | 0.525242372030153 |
| 84 | PROGRAM SEMANTICS AND VERIFICATION | AI | UU | 3.13160426380718e-05 | 0.288205654148651 | 0.000263743259052266 | 0.660118472821975 | 3.36028134899757e-05 | 0.000341129257664616 | 0.0510060816565296 |
| 85 | TECHNOLOGIES FOR LEARNING | AI | UU | 0.458345397038962 | 0.000226478268470014 | 0.0295100784359423 | 0.000146362823660075 | 0.000632016821154072 | 0.119164130374817 | 0.391975536236995 |
| 86 | PROBABILISTIC REASONING | AI | UU | 0.000295818434535261 | 0.000312216532116297 | 0.156982758288295 | 0.189334509374143 | 0.000307131600024482 | 0.493445898584676 | 0.159321667186209 |
| 87 | BIG DATA | AI | UU | 7.24369204989819e-06 | 0.233950096290598 | 0.000121989397120521 | 0.74500513337821 | 1.20230513501809e-05 | 0.000139067155692483 | 0.0207644470349792 |
| 88 | PATTERN SET MINING | AI | UU | 0.000284380364831384 | 0.569048528715236 | 0.239929952588797 | 0.0408237028599904 | 7.54217314924876e-05 | 6.12062484271647e-05 | 0.149776807491226 |
| 89 | MULTIMEDIA RETRIEVAL | AI | UU | 0.00054099474345016 | 0.000390957739006521 | 0.284932752606414 | 0.118779003482757 | 0.358381311850207 | 0.000189593559801692 | 0.236785386018364 |
| 90 | COMPUTER VISION | AI | UU | 0.000316230538210634 | 0.964116631406259 | 0.000177555267567789 | 0.000752235353907196 | 1.61874310598068e-05 | 0.00020672293012745 | 0.0344144370728684 |
| 91 | CROWD SIMULATION | AI | UU | 0.026570953982789 | 0.000447373181998905 | 0.000450532571538203 | 0.001445528091385 | 0.222165775998021 | 0.0485774306310456 | 0.700342405543222 |
| 92 | ADVANCED COGNITIVE AND SOCIAL PSYCHOLOGY FOR HCI | AI | UU | 0.00818935012901484 | 0.000637047185367076 | 0.000322049447088528 | 0.00188441483905641 | 0.105648005090503 | 0.154087338933632 | 0.729231794375338 |
| 93 | ADAPTIVE INTERACTIVE SYSTEMS | AI | UU | 0.00380405818567252 | 0.000609268070591322 | 3.63334274312724e-05 | 0.00135778260859692 | 0.000571010116321562 | 0.722623851075974 | 0.270997696515412 |
| 94 | MEANINGFUL (LINKED) DATA INTERACTION | AI | UU | 0.223360907216158 | 0.000475176393431496 | 0.328665221606389 | 0.000167462135793495 | 0.000517977762032016 | 0.000287001649139986 | 0.446526253237055 |
| 95 | NATURAL LANGUAGE GENERATION | AI | UU | 0.0855598330797627 | 0.000220821142267478 | 0.209560225578532 | 0.000352414152095207 | 0.235635850488696 | 0.000430813502341881 | 0.468240042056305 |
| 96 | APPLIED COGNITIVE PSYCHOLOGY RESEARCH TOOLBOX | AI | UU | 0.137995860163516 | 0.0702439261787882 | 0.000648979653633587 | 0.000679114253341815 | 0.000487048799637326 | 0.0155488695578797 | 0.774396201393204 |
| 97 | FOUNDATIONS OF SOUND PATTERNS | AI | UU | 0.0204550082780928 | 0.00541289251725716 | 0.0034420167873721 | 0.00460970047649282 | 0.00257730621327214 | 0.00821667538013169 | 0.955286400347381 |
| 98 | REASONING ABOUT MEANING IN LINGUISTIC COMMUNICATION | AI | UU | 0.0844510124091312 | 0.000936833762902432 | 0.157461280448265 | 0.000551930800407001 | 0.000746909516571489 | 0.000808418960592969 | 0.75504361410213 |
| 99 | COGNITIVE AND COMPUTATIONAL ASPECTS OF WORD MEANING | AI | UU | 0.00479398259158237 | 0.0300997511159833 | 0.405352419897712 | 0.00271852472705951 | 0.000609382783185947 | 0.000415496372109957 | 0.556010442512367 |
| 100 | TOPICS IN PHILOSOPHY OF MIND | AI | UU | 0.013959626556682 | 0.00240565410349079 | 0.000321833477844709 | 0.031593355248348 | 0.000657091138624509 | 0.12273727025598 | 0.82832516921903 |
| 101 | DIGITAL ETHICS | AI | UU | 0.312854871226424 | 3.31971085708329e-05 | 2.43440293521644e-05 | 5.67941957857585e-05 | 0.217243305118496 | 0.247777970762973 | 0.222009517558398 |
| 102 | TOPICS IN EPISTEMOLOGY AND PHILOSOPHY OF SCIENCE | AI | UU | 0.280424354368024 | 0.000618982649524725 | 0.000131274710040956 | 0.000261057380987573 | 0.000695293783913297 | 0.174453448182515 | 0.543415588924995 |
| 103 | SOCIAL AND AFFECTIVE NEUROSCIENCE | AI | UU | 0.0163137097131193 | 0.00190233105883535 | 0.00184052401141341 | 0.00169143806630847 | 0.00125684522032321 | 0.00351391751315577 | 0.973481234416845 |
| 104 | NEUROCOGNITION OF MEMORY AND ATTENTION | AI | UU | 0.0135749818894596 | 0.00296435352743428 | 0.00235880796038232 | 0.00314598491008151 | 0.00181874844916431 | 0.00624662760568423 | 0.969890495657794 |
| 105 | PHILOSOPHY OF NEUROSCIENCE | AI | UU | 0.0909346915425691 | 0.00150209096126438 | 0.00155444445936193 | 0.000801651743389734 | 0.0011191303419349 | 0.00361441884090797 | 0.900473572110572 |
| 106 | BASIC FMRI ANALYSIS | AI | UU | 0.00504364628170916 | 0.492620280189711 | 0.000963070618002735 | 0.00125412853564864 | 0.000217499303775569 | 0.00142736473879498 | 0.498474010332358 |
| 107 | REQUIREMENTS ENGINEERING | AI | UU | 0.00172048721382149 | 0.000767000818075832 | 0.918965204452416 | 0.00085929188638399 | 0.000466100986263556 | 2.92231605896009e-05 | 0.0771926914824496 |
| 108 | SOFTWARE ARCHITECTURE | AI | UU | 0.055079109564277 | 0.000210001024880118 | 0.0160732413627818 | 0.000379939038211903 | 0.558098822687559 | 0.00100887643673028 | 0.36915000988556 |
| 109 | BUSINESS INTELLIGENCE | AI | UU | 0.00194290776232689 | 0.000353208828680734 | 0.000948611597596499 | 0.0309541800803236 | 0.718577211191059 | 0.00250778172111149 | 0.244716098818902 |
| 110 | METHOD ENGINEERING | AI | UU | 0.000253618213211356 | 0.000161759831199539 | 0.000428442742520511 | 0.181630489301306 | 0.726644145604109 | 0.00112744850324844 | 0.0897540958044051 |
