# Supplementary material for: Unravelling the skills of data scientists: A text mining analysis of Dutch university master programs in data science and artificial intelligence
Source: PLoS One. 2024 Feb 29;19(2):e0299327. doi: 10.1371/journal.pone.0299327 (PMC10903789; doi:10.1371/journal.pone.0299327)
Supplement: Attachment — Submitted filename: Response to Reviewers.docx [file pone.0299327.s007.docx]

Dear Editor,

We would like to thank you and the reviewers for the very useful comments. I will show below in detail after each comment how we addressed the comment in the manuscript using italic, and provide citations from the revised manuscript where relevant.

Kind regards,

The authors

Dear Authors,

Subject: Manuscript Decision – Major Revision Required

Following a thorough evaluation of your manuscript, which applies an innovative methodology to study the skillset of data science Master's students in Dutch higher education institutions, the decision is that a major revision is required prior to considering its publication. Your article presents a timely and insightful contribution, with quality writing and structure, referencing relevant literature, and providing insightful findings with practical implications. However, I have identified several issues that need to be addressed to enhance the clarity and coherence of the work.

1) Inconsistency in the Number of Institutions: On pages 2 and 15, 7 Dutch universities are mentioned, while on page 10, 11 institutions and a collaboration of 3 institutions are referred to. It is important to clarify the exact number of higher education institutions included in the sample and ensure this information is consistent throughout the article.

*We would like to thank you for pointing out this inconsistency. Indeed we have 11 universities in the sample 1 plus an MSc program that is organized in cooperation between 3 universities as a joint program. We now corrected this error in the abstract and on page 15.*

2) Clarification of the Third Research Question (RQ3): I request a clarification on what is meant by "general" vs. "subject-specific" data science programs. Providing examples or further explanation of this distinction would be helpful.

*Thank you for this comments. Indeed the differentiation was not clearly specified- we meant by general programs data science programs that are not field specific, and by subject-specific programs, programs that focus on data science within a disciplinary context for example Data science for marketing, or for the social sciences. – we now add an explanation on page 4 (see also citation below), and in the new appendix D in the last column we provide the classification for each master program.*

“(3) How do the skillsets learned at more general data science and AI programs differ from those learned at more subject-specific data science master programs? In this context, we define 'general data science and AI programs' (G) as programs exclusively focused on this domain. We refer to 'subject-specific programs' (S) as those that concentrate on domain-specific applications of data science, such as data science for marketing.”

*Furthermore, we also discuss this in more detail in the results section, where the new Figure 3 zooms in more specifically on the distribution of the 7 topics over these two program types, see page 13:*

“To address Research Question 3, we examined the distribution of posterior classifications by program type, as depicted in Figure 3, distinguishing between general programs—those with a broad focus on data science and AI skills—and subject-specific programs, which concentrate on a more narrow, domain-specific application of data science, such as in life sciences or marketing.

[Figure 3 about here]

Figure 3 illustrates a pronounced distinction between the two types of programs. General programs tend to score higher in the data processing clusters, particularly in research and electronics/IT. Conversely, subject-specific programs predominantly feature core terms related to statistics and ethics. A closer examination of the defining words within these clusters reveals that subject-specific programs not only emphasize data science technical skills but also integrate terms associated with ethics, health, and business. In contrast, general programs are characterized by a focus on technical and engineering skill sets.”

*Finally, we now also refer more explicitly to the findings of Research Question 3 in the abstract and discussion and conclusion section of the article.*

3) Use of Terms "Skills", "Competencies", "Knowledge": These terms are interchangeably used but do not exactly mean the same thing. I recommend choosing one term to use consistently and defining it clearly.

*We agree with the reviewer that we used these terms loosely. The reason for this is that because we extracted the key terms directly from the data using unsupervised clustering some of the key terms are skills, while others fall under competencies or knowledge domains. We explicitly explain this issue in the current version on page 4:*

“In this context, we define 'general data science and AI programs' (G) as programs exclusively focused on this domain. We refer to 'subject-specific programs' (S) as those that concentrate on domain-specific applications of data science, such as data science for marketing. Moreover, it is important to clarify that our methodology employs unsupervised clustering combined with text mining techniques to derive a categorization of skills from the descriptions of the programs and courses. This approach ensures that the resulting clusters encapsulate a comprehensive range of knowledge, skills, and competencies (KSC), as characterized by Heller, Unlu, and Albert (2013). Our objective is to provide a holistic understanding of the KSCs imparted by these master's programs, thereby refraining from distinguishing between KSCs. For the sake of brevity and clarity in the remainder of this article, we will refer collectively to these elements as 'skills', implicitly encompassing the full spectrum of KSCs as discussed.”

*We use “skills” thereafter as the only general term. The term skill set is used when referred to a set of skills.*

4) Inclusion of Institutions from the VSNU Group: On page 10, it is mentioned that only institutions that are part of the VSNU group were included for reliability reasons. Please explain what this means, how this group compares to other higher education institutions, and how representative it is of the educational system.

*We appreciate the reviewer's request for clarification regarding the inclusion of institutions from the VSNU group. The VSNU, now known as 'The Universities of the Netherlands', is a consortium recognized by the Dutch government that comprises all major government-funded universities in the Netherlands. This includes ten research universities, three specialized institutions, and one open university, which collectively represent 74% of the country's research universities. Membership in the VSNU assures adherence to stringent quality benchmarks and uniform academic standards across these institutions.*

*The excluded 26% of research universities consist of five small, specialized institutions: three theological universities dedicated to theological studies for clergy, one military university focused on defense education, and one private business school. Notably, these institutions do not offer master's programs in data science or artificial intelligence, which are the focus of our study.*

*To maintain the focus on the research scope and to avoid potential confusion, we have omitted the detailed information about the VSNU in our revised manuscript. We have instead emphasized that our analysis encompasses all Dutch master's programs in data science and AI for which course and/or program descriptions were publicly available, ensuring the reliability of our results. This refinement clarifies that the study is comprehensive within the context of the relevant educational programs in the Netherlands.*

5) Addition of Information in the Annex: It would be beneficial to include a table in the Annex listing the exact names/titles of the data science programs per institution. Additionally, understanding the size of the corpus for each institution would be useful.

*We added an extra table with this info into the new appendix D that shows all programs per university and the size of the corpus for the academic year 2020/21, the year of data collection. This table also has a column, that shows if a program was classified as general Data science/ AI (G) or domain specific (S) that helps interpreting the results for RQ3.*

6)*Inclusion of "Soft Skills": I would like to know what your findings suggest about the inclusion of "soft skills" in Dutch data science Master's programs.

*Thank you for bringing this up, to motivate us to further elaborate. We actually found it concerning how using the text mining approach soft skills did not surface at all among the skills that define the clustering. We did a data driven approach- so this means this skills are not central enough/ not present to inform the clustering. We suggest for follow up research to look into more details about why these skills are not central. Follow up research should look into this very important issue in more detail, using different approaches- for example by interviewing program coordinators, because the course descriptions in their own did not provide sufficient data on this very important topic. We specifically discuss the problem in the discussion on page 14:*

*“*A notable finding from our analysis is furthermore the absence of soft skills in the clustering results. Neither the 7-topic nor the more comprehensive 13-topic solutions revealed any reference to soft skills within the curricula. This observation underscores that soft skills are not a central or frequent component in the master's programs we examined. While this finding aligns with previous research, which also highlights a predominant focus on technical skills (Tang and Sae-Lim, 2016; West, 2017; Cegielksi and Jones-Farmer, 2016), it remains a concerning trend. Nonetheless, this trend is concerning, particularly when juxtaposed with research highlighting the demand for soft skills in the job market, underscoring their significance (Allen et al., 2021; Mauro et al., 2017; Markow et al., 2017). In light of this discrepancy between curricular content and market needs, we recommend further investigation, possibly through qualitative methods such as interviews with program managers, to explore the reasons behind this lack of emphasis on soft skills.*”*

Please consider these points and make the necessary revisions. I am available for any further clarifications that may be required during the revision process.

Sincerely,

==============================

Please submit your revised manuscript by Dec 30 2023 11:59PM. If you will need more time than this to complete your revisions, please reply to this message or contact the journal office at [plosone@plos.org](mailto:plosone@plos.org). When you're ready to submit your revision, log on to <https://www.editorialmanager.com/pone/> and select the 'Submissions Needing Revision' folder to locate your manuscript file.

Please include the following items when submitting your revised manuscript:

- A rebuttal letter that responds to each point raised by the academic editor and reviewer(s). You should upload this letter as a separate file labeled 'Response to Reviewers'.
- A marked-up copy of your manuscript that highlights changes made to the original version. You should upload this as a separate file labeled 'Revised Manuscript with Track Changes'.
- An unmarked version of your revised paper without tracked changes. You should upload this as a separate file labeled 'Manuscript'.

If you would like to make changes to your financial disclosure, please include your updated statement in your cover letter. Guidelines for resubmitting your figure files are available below the reviewer comments at the end of this letter.

If applicable, we recommend that you deposit your laboratory protocols in protocols.io to enhance the reproducibility of your results. Protocols.io assigns your protocol its own identifier (DOI) so that it can be cited independently in the future. For instructions see: [https://journals.plos.org/plosone/s/submission-guidelines#loc-laboratory-protocols](https://eur03.safelinks.protection.outlook.com/?url=https%3A%2F%2Fjournals.plos.org%2Fplosone%2Fs%2Fsubmission-guidelines%23loc-laboratory-protocols&data=05%7C01%7Cz.bakk%40fsw.leidenuniv.nl%7C66873010be7d4f6c5c0208dbe6274c8d%7Cca2a7f76dbd74ec091086b3d524fb7c8%7C0%7C0%7C638356828821669604%7CUnknown%7CTWFpbGZsb3d8eyJWIjoiMC4wLjAwMDAiLCJQIjoiV2luMzIiLCJBTiI6Ik1haWwiLCJXVCI6Mn0%3D%7C3000%7C%7C%7C&sdata=d1eauwoeEk%2BWwT9jSdf3V9WUUVAAJy%2Fo9sfRQ%2BqVTqk%3D&reserved=0). Additionally, PLOS ONE offers an option for publishing peer-reviewed Lab Protocol articles, which describe protocols hosted on protocols.io. Read more information on sharing protocols at [https://plos.org/protocols?utm_medium=editorial-email&utm_source=authorletters&utm_campaign=protocols](https://eur03.safelinks.protection.outlook.com/?url=https%3A%2F%2Fplos.org%2Fprotocols%3Futm_medium%3Deditorial-email%26utm_source%3Dauthorletters%26utm_campaign%3Dprotocols&data=05%7C01%7Cz.bakk%40fsw.leidenuniv.nl%7C66873010be7d4f6c5c0208dbe6274c8d%7Cca2a7f76dbd74ec091086b3d524fb7c8%7C0%7C0%7C638356828821669604%7CUnknown%7CTWFpbGZsb3d8eyJWIjoiMC4wLjAwMDAiLCJQIjoiV2luMzIiLCJBTiI6Ik1haWwiLCJXVCI6Mn0%3D%7C3000%7C%7C%7C&sdata=Q%2BpdYbS5tjebVlaEl%2FedMsUQtJpfWwmx6E9pnc1lKAM%3D&reserved=0).

We look forward to receiving your revised manuscript.

Kind regards,

Ricardo Limongi
Academic Editor
PLOS ONE

Journal requirements:

When submitting your revision, we need you to address these additional requirements.
