# Supplementary material for: Unravelling the skills of data scientists: A text mining analysis of Dutch university master programs in data science and artificial intelligence
Source: PLoS One. 2024 Feb 29;19(2):e0299327. doi: 10.1371/journal.pone.0299327 (PMC10903789; doi:10.1371/journal.pone.0299327)
Supplement: Attachment — Submitted filename: replytoreviewersR2_SubmissionPlosone.docx [file pone.0299327.s008.docx]

Dear Zuzsa Bakk,

Recommendations for Revisions - Unravelling the Skill Sets of Data Scientists:  A Text Mining Analysis of Dutch University Master Programs in Data Science and Artificial Intelligence

Your manuscript, examining similarities and differences across master programs in data science and artificial intelligence in Dutch universities, presents an insightful and timely contribution to the field. The novel methodology applied to study the skillset of data science Master's students in Dutch higher education institutions is commendable. The manuscript is well-written and well-structured, with relevant literature references and insightful findings. However, to strengthen the manuscript for potential publication, I recommend addressing the following points:

1. The assertion that the growth in data science programs has been driven more by market needs than theoretical development warrants a more nuanced discussion. Consider incorporating viewpoints that acknowledge the significant role of theoretical advancements in the field, such as large language models and generative adversarial networks, in driving the evolution of research and educational programs in data science.

We would like to thank the reviewer for highlighting this point. We agree that the underlying processes are both driven by both theoretical and market developments. We clarify now this better in the introduction.

“These market changes are strongly driven by theoretical developments, namely the development of large language models (Usman Hadi et al., 2023) and generative adversarial networks (Creswell et al., 2018). These new modeling developments have also led to a need to adjust traditional data driven master programs to encompass these new theoretical developments, and to integrate theoretical knowledge into interdisciplinary master programs. The rapid development of these new modeling approaches represents a continuous challenge to data science master programs to adopt their curricula.”

And also nuance the statement in the theory section:

“This growth has been driven by a combination of increasing market needs and theoretical development of the field of data science, as well as related fields such as business analytics or big data. Yet, the definition of the field is often driven by market needs and technology forecasts (for example Gorman & Kimberg, 2014). ”

2. Enhance the depth of your results section. It is crucial to delve deeper into the implications of findings, such as the omnipresence of the word “data” in all topics. Clarify the significance of such observations in differentiating and classifying courses. Additionally, it provides a clearer context, explanation, and discussion for the posterior distributions and their means, emphasizing their relevance in the distribution of topics across universities.

We agree, and are thankful for this suggestion, indeed the word data is present in almost all clusters. That means that it is a very widely use word- and while overly relevant in all topics, less differentiating between clusters. We discuss the word clusters in more detail now in the result section:

“When we look at the indicated clusters of skills in Table 3, we can clearly see that there is a wide variety of skills needed to complete a data science master program in the Netherlands. At the same time, there is also some overlap between the 7 topics. Most predominantly, the word “data” is present in every topic, not surprisingly, given its central role in these master programs. Also the words “model” (present in topics 3, 4, 5, 7), “Methods” (topics 3, 4, 7) and “machine learning” (topics 3, 4) are central to the clustering. Their presence in all these different clusters shows the wide spread importance of these skills. Each topic, however, has a specific focus characterized by the composition of the most frequent 10 words. For example, topic 2 is described by words like “statistical”, “theory”, “linear”, “data analysis”- that relate more to the more traditional statistical aspects of data science. In contrast, topic 3 focuses on “deep learning”, “natural language processing”, “machine learning”- more innovative elements of a broader data science field, while topic 5 encompasses skills like “health”, “ethical”, “decision”, pointing to applications of data science in different domains. We manually labelled each topic based on the 10 most frequent words and distinguished between core elements of data science and subdomains in Table 4 to aid interpretation.”

Furthermore with regard to the posterior distribution per universities we now updated figure 3, making it also colored, hopefully this improves the ease of readability.

3. Expand the discussion to include a more detailed analysis of individual courses, considering they form the basic unit of your study. Investigate within-group variability, such as similarities and differences among courses and programs within the same university and how they compare to those between different universities. Incorporate hierarchical clustering analysis at multiple levels (courses, programs, universities) to provide a more comprehensive understanding.

We thank the reviewer for this suggestion. We have added now a more detailed discussion of the 2 programs at Utrecht University and the two programs at Groningen university with the distribution of topics per program. We visualize this in Appendix E, and discuss it in the result section (see citation below). For this 4 programs we also add in Appendix F the per course distribution of the topics, while for all the courses this data is available in our R environment that we make available at OSF open publication of the materials.

“Even within the same university, there can be large differences between skills taught, depending on the focus of the master programs. For illustrative purposes, Appendix E presents the posterior distribution of the seven topics across the two master programs offered by Utrecht and Groningen University. At Utrecht University, both the Applied Data Science and Artificial Intelligence programs have a similar focus on the research pillar. However, the Applied Data Science program has a heavier focus on core terms and statistics than the Artificial Intelligence program. At the same time, at Groningen University, the specialized Marketing Analytics and Data Science program shows less emphasis on the second to forth pillars than the general Artificial Intelligence program. Interestingly, the two AI master programs at these universities are more similar to each other than to the two more applied, specialized programs, and this pattern is also observed in the subject specific programs.

To address Research Question 3, we examined the distribution of posterior classifications by program type, as depicted in Figure 3. This analysis distinguishes between general programs—with a broad focus on data science and AI skills—and subject-specific programs, which concentrate on a more narrow, domain-specific application of data science, such as in life sciences or marketing. Similar to the more detailed comparison in Appendix E, Figure 3 also shows a marked difference between general programs, which focus on the more technical pillars 3-5, and the subject-specific programs, which stand out in the first two, and last research pillars, thus the less technical pillars. “

We are thankful for the suggestion of using a multilevel model, because at first instance we also considered the idea of modeling the multilevel structure in the data, however that was not possible for several reasons: namely *i.* lack of a large enough sample at university level (only 11 higher level units), and *ii* most importantly lack of proper multilevel models for clustering with unsupervised text mining using Latent variables for interpretable topics).

We carefully checked other manuscripts using similar unsupervised text mining approaches to educational data (West, 2017 ) and also clustering for job market data (Verma et al, 2019, 2022, Almgerbi et al., 2022) and none of them applied a multilevel approach- because of a lack of such an approach. Also studies that applied a more qualitative approach ignore the multilevel structure, due to the complexity of properly analyzing it (Lee and Delaney, 2021, Gorman and Klimberg, 2014, Tang and Sae-Lim,2016). The reason for the lack of such proper multilevel models for these type of data probably lies in the sparsity of the word by document matrix that is used for the clustering. How to fit a multilevel structure on such sparse data is not obvious. We now added a paragraph in the Discussion where we clearly mention the lack of hierarchical approaches that are similar to multilevel regression in the field of multidimensional data reduction using unsupervised text mining approaches:

“Another shortcoming of our approach is that although the data has a multilevel structure, with courses and programs nested within universities, we did not explicitly model this multilevel structure. The reason for this is that currently no topic modelling approach is available that can properly model the hierarchical structure in the data. ”

Furthermore we summarize basic descriptive statistics about the individual courses and programs in Table 1, where the number of programs and separately core and specialization courses per university are provided, and in appendix D we also provide the number of students per program. Appendix D gives a bit more insights about the variability with regard to the size of the programs as well, showing their importance on the market.

4. Adjust the figures and their fonts to enhance readability. This is essential for you to convey your findings and facilitate reader comprehension.

We adjusted the figures. Figure 2 is now color and we changed the setup from having multiple barcharts stacked next to each other, to a single bar chart- so space is not wasted for showing the axis multiple times. Adding color to the graph also helps showing the differences between the universities. Furthermore we changed Figure 3 from a bar chart to a line-chart, because this occupies less space and highlights the differences even better. In appendix E we also added a new lien chart about the 2 programs at UU, and the 2 programs at RUG.

5. Conduct a thorough spell check and grammar review. Address typographical errors like the one found in the first paragraph of section 3 (“correlated”).

Thank you for this comment. We revised the ms for grammar.

6. Clarify inconsistencies in the number of Dutch universities included in the study. Could you make sure uniformity in the number of institutions referenced throughout the paper?

*We would like to thank you for pointing out this inconsistency. We have 11 universities in the sample plus an MSc program that is organized in cooperation between 3 universities as a joint program. We now corrected this error in the abstract and on page 15.*

6. Refine your third research question to clearly distinguish between "general" and "subject-specific" data science programs. Providing specific examples or a more detailed explanation would enhance clarity.

*Thank you for this comments. Indeed the differentiation was not clearly specified- we meant by general programs data science programs that are not field specific, and by subject-specific programs, programs that focus on data science within a disciplinary context for example Data science for marketing, or for the social sciences. – we now add an explanation on page 4 (see also citation below), and in the new appendix D in the last column we provide the classification for each master program.*

“(3) How do the skills learned at more general data science and AI programs differ from those learned at more subject-specific data science master programs? In this context, we define 'general data science and AI programs' (G) as programs exclusively focused on this domain. We refer to 'subject-specific programs' (S) as those that concentrate on domain-specific applications of data science, such as data science for marketing. ”

*Furthermore, we also discuss this in more detail in the results section, where the new Figure 3 zooms in more specifically on the distribution of the 7 topics over these two program types, see page 13:*

“To address Research Question 3, we examined the distribution of posterior classifications by program type, as depicted in Figure 3. This analysis distinguishes between general programs—with a broad focus on data science and AI skills—and subject-specific programs, which concentrate on a more narrow, domain-specific application of data science, such as in life sciences or marketing. Similar to the more detailed comparison in Appendix E, Figure 3 also shows a marked difference between general programs, which focus on the more technical pillars 3-5, and the subject-specific programs, which stand out in the first two, and last research pillars, thus the less technical pillars.

|  | General | Subject specific |
| --- | --- | --- |
| Core terms | 0.121765 | 0.189307 |
| Statistics | 0.117567 | 0.162392 |
| Data proc_3 | 0.114424 | 0.0848597 |
| Data proc_4 | 0.176228 | 0.0895854 |
| Electronics/IT | 0.144272 | 0.110259 |
| Ethics | 0.0945531 | 0.151788 |
| Research | 0.231189 | 0.211809 |

Figure 3 The posterior distribution per program type (general vs subject specific) of the 7 topics of the CTM model. The classification of programs into general vs. subject specific are available in Appendix D.

Figure 3 illustrates a pronounced distinction between the two types of programs. General programs tend to score higher in the data processing clusters, particularly in research and electronics/IT. Conversely, subject-specific programs predominantly feature core terms related to statistics and ethics. A closer examination of the defining words within these clusters reveals that subject-specific programs not only emphasize data science technical skills but also integrate terms associated with ethics, health, and business. In contrast, general programs are characterized by a focus on technical and engineering skills.”

7. Standardize the terminology used throughout the paper. You can choose a consistent term (skills, competencies, knowledge) and give a clear definition to avoid ambiguity.

*We agree with the reviewer that the terms should be better standardized. We extracted the key terms directly from the data using unsupervised clustering some of the key terms are skills, while others fall under competencies or knowledge domains. We explicitly explain this issue in the current version on page 4:*

“Moreover, it is important to clarify that our methodology employs unsupervised clustering combined with text mining techniques to derive a categorization of skills from the descriptions of the programs and courses. This approach ensures that the resulting clusters encapsulate a comprehensive range of knowledge, skills, and competencies (KSC), as characterized by Heller, Unlu, and Albert (2013). These clusters are not intended to segregate the three dimensions of KSCs; rather, we use the term 'skills' as an inclusive term that embodies all three dimensions. Our objective is to provide a holistic understanding of the KSCs imparted by these master programs, thereby refraining from distinguishing between knowledge, skills, and competencies. For the sake of brevity and clarity in the remainder of this article, we will refer collectively to these elements as 'skills', implicitly encompassing the full spectrum of knowledge, skills, and competencies as discussed. “

*We use “skills” thereafter as the only general term.*

9. Elaborate on the inclusion criteria for the VSNU group of institutions. Could you clarify how these institutions compare to others and the representativeness of your sample?

*We appreciate the reviewer's request for clarification regarding the inclusion of institutions from the VSNU group. The VSNU, now known as 'The Universities of the Netherlands', is a consortium recognized by the Dutch government that comprises all major government-funded universities in the Netherlands. This includes ten research universities, three specialized institutions, and one open university, which collectively represent 74% of the country's research universities. Membership in the VSNU assures adherence to stringent quality benchmarks and uniform academic standards across these institutions.*

*The excluded 26% of research universities consist of five small, specialized institutions: three theological universities dedicated to theological studies for clergy, one military university focused on defense education, and one private business school. Notably, these institutions do not offer master's programs in data science or artificial intelligence, which are the focus of our study.*

*To maintain the focus on the research scope and to avoid potential confusion, we have omitted the detailed information about the VSNU in our revised manuscript. We have instead emphasized that our analysis encompasses all Dutch master's programs in data science and AI for which course and/or program descriptions were publicly available, ensuring the reliability of our results. This refinement clarifies that the study is comprehensive within the context of the relevant educational programs in the Netherlands.*

10. Add an annex listing the specific titles of data science programs per institution. Include the size of the corpus for each institution, which would enrich the context of your analysis.

*We added an extra table with this info into the new appendix D that shows all programs per university and the size of the corpus for the academic year 2020/21, the year of data collection. This table also has a column, that shows if a program was classified as general Data science/ AI (G) or domain specific (S) that helps interpreting the results for RQ3.*

11. Explore the inclusion of "soft skills" in Dutch data science Master's programs and discuss the implications of your findings in this regard.

*Thank you for bringing this up, to motivate us to further elaborate. We actually found it concerning how using the text mining approach soft skills did not surface at all among the skills that define the clustering. We did a data driven approach- so this means this skills are not central enough/ not present to inform the clustering. We suggest for follow up research to look into more details about why these skills are not central. Follow up research should look into this very important issue in more detail, using different approaches- for example by interviewing program coordinators, because the course descriptions in their own did not provide sufficient data on this very important topic. We specifically discuss the problem in the discussion on page 16-17:*

“A notable finding from our analysis is furthermore the absence of soft skills in the clustering results. Neither the 7-topic nor the more comprehensive 13-topic solutions revealed any reference to soft skills within the curricula. This suggests that soft skills are not a central or frequent component in the master programs we examined. While this finding aligns with previous research, which also highlights a predominant focus on technical skills (Tang and Sae-Lim, 2016; West, 2017; Cegielksi and Jones-Farmer, 2016), it remains a concerning trend, especially considering research that highlights the demand for soft skills in the job market (Allen et al., 2021; Mauro et al., 2017; Markow et al., 2017). In light of this discrepancy between curricular content and market needs, further investigation, possibly through qualitative methods such as interviews with program managers, is recommended to explore the reasons behind this lack of emphasis on soft skills.”

Your manuscript offers valuable insights into the field of data science education. I think addressing these recommendations will enhance your work's clarity, coherence, and overall impact. We are excited to receive your revised manuscript.

Sincerely,

***
